# Prediction of Protein Structural Class Based on Gapped-Dipeptides and a Recursive Feature Selection Approach

**DOI:** 10.3390/ijms17010015

**Published:** 2015-12-24

**Authors:** Taigang Liu, Yufang Qin, Yongjie Wang, Chunhua Wang

**Affiliations:** 1 College of Information Technology, Shanghai Ocean University, Shanghai 201306, China; tgliu@shou.edu.cn (T.L.); yfqin@shou.edu.cn (Y.Q.); 2 College of Food Science & Technology, Shanghai Ocean University, Shanghai 201306, China

**Keywords:** feature selection, gapped-dipeptide, position-specific score matrix, protein structural class, recursive feature elimination, support vector machine

## Abstract

The prior knowledge of protein structural class may offer useful clues on understanding its functionality as well as its tertiary structure. Though various significant efforts have been made to find a fast and effective computational approach to address this problem, it is still a challenging topic in the field of bioinformatics. The position-specific score matrix (PSSM) profile has been shown to provide a useful source of information for improving the prediction performance of protein structural class. However, this information has not been adequately explored. To this end, in this study, we present a feature extraction technique which is based on gapped-dipeptides composition computed directly from PSSM. Then, a careful feature selection technique is performed based on support vector machine-recursive feature elimination (SVM-RFE). These optimal features are selected to construct a final predictor. The results of jackknife tests on four working datasets show that our method obtains satisfactory prediction accuracies by extracting features solely based on PSSM and could serve as a very promising tool to predict protein structural class.

## 1. Introduction

Proteins can perform many biological functions within living organisms when they fold and take on a three-dimensional structure [1,2,3,4]. According to the concept of structural class introduced by Levitt and Chothia [5], proteins are divided into four major structural classes: all-α, all-β, α/β and α + β. The knowledge of protein structural class can provide important and useful information about a protein’s three-dimensional structure and its functionality [6]. However, it is usually time-consuming and costly to determine the structure information of a protein by just relying on wet-bench experiments. On the other hand, sequence information has grown exponentially with the help of high-throughput sequencing techniques, which has made a huge gap between the sequence and structure space. Hence, there is a great need to explore bioinformatics prediction methods based on sequence data to fill this gap.

From the pattern recognition perspective, predicting protein structural class is usually described as a multi-class classification problem. During the past 30 years, various significant efforts have been made to solve this problem. These methods generally consist of two major steps: (1) protein sequence representation or feature extraction; (2) algorithm selection for classification. Many classification techniques have been proposed to perform the prediction of protein structural class such as neural network [7], support vector machine (SVM) [8,9,10], fuzzy *k*-nearest neighbor [11,12], fuzzy clustering [13], Bayesian classification [14], Logistic regression [15,16], rough sets [17], and ensembles of classifiers [18,19,20,21,22]. Among these algorithms, SVM has attained the best prediction performance for this task [9]. At the same time a wide range of sequence features have been used to reveal more discriminatory information for protein structural class, including amino acid composition (AAC) [23,24], pseudo-AAC [25,26,27], position-specific score matrix (PSSM) profile [28,29,30,31] and predicted secondary structure [32,33,34]. As a powerful feature extraction tool for analyzing DNA or protein sequences, pseudo-AAC has been widely applied to the field of bioinformatics [35,36,37,38,39,40].

Among the above sequence features, the most significant enhancements in prediction accuracy are based on the PSSM profile and predicted secondary structure. Since the prediction performance of protein secondary structure using PSIPRED software [41] crucially relies on PSSM, the PSSM profile provides more important and original discriminatory information for protein structural class prediction. Recently, several methods have been developed to extract the potential local and global information from PSSM such as AAC [31], dipeptide composition [31], auto covariance (AC) [30], and linear correlation coefficient [29]. However, the informative features encoded in PSSM have not been adequately explored due to limited prediction accuracy. This highlights the need for exploring more effective feature extraction techniques to represent protein sequences.

In this study, we introduce a feature extraction approach based on gapped-dipeptides (*i.e.*, two residues separated by one or more positions) composition (GapDPC) to further explore more discriminatory information solely from the PSSM profile. The processes of our method are as follows. First, the PSSM profile of a protein is transformed into a fix-length feature vector by extracting GapDPC. Then, a recursive feature selection approach is applied to reduce feature redundancy and optimal features are input to an SVM classifier to conduct the prediction. Finally, validation results on four working datasets indicate that our method presents outstanding improvements in prediction accuracies compared with other existing methods.

## 2. Results and Discussion

### 2.1. Parameter Selection

**Figure 1:**
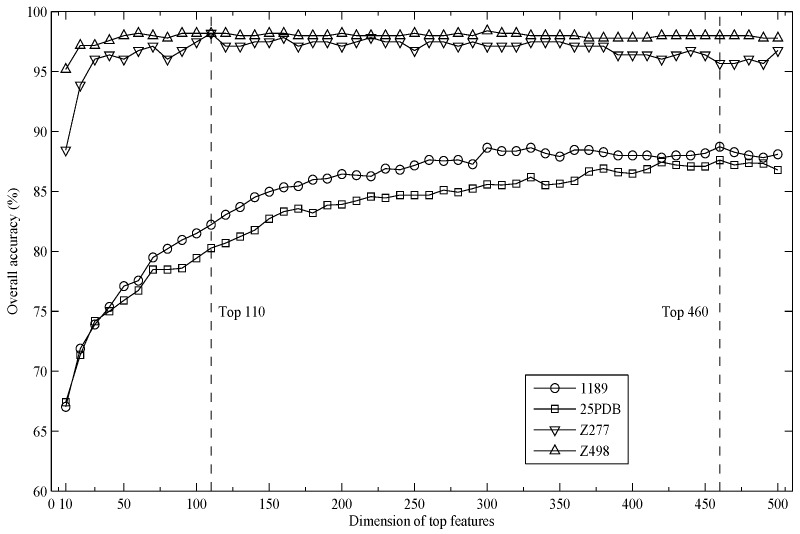
This graph shows how different top *K* features affect the overall accuracies.

Preliminary test results indicate that the length of the shortest sequence in the dataset is 10. By integrating GapDPC with different gapped distances, the value of parameter *G* is set to eight in this study, which results in 3600 features for each protein sequence. Then, these features are ranked based on their relevance to sample classification by support vector machine-recursive feature elimination (SVM-RFE). To explore the impact of selected feature dimensions on prediction performance, we calculate the overall accuracies for top *K* features using five-fold cross-validation, where *K* = 10, 20, 30, ... , 500. The results are shown in Figure 1. As can be seen, the overall accuracies for the 1189 and 25PDB datasets achieve a maximum value when *K* increases to 460. Thus, the top 460 features are selected to further compute the accuracies for two low-similarity datasets by jackknife tests. Similarly, the top 110 features are adopted for two small datasets, Z277 and Z498, due to their high accuracies. The results of jackknife tests on four datasets are listed in Table 1.

**Table 1:** Prediction performances on four datasets by our method.

Dataset	Accuracy (%)					Matthews Correlation Coefficient (MCC)			
	All-α	All-β	α/β	α + β	Overall	All-α	All-β	α/β	α + β
Z277	97.1	98.4	97.5	96.9	97.5	0.96	0.98	0.97	0.96
Z498	98.1	100	98.5	97.7	98.6	0.96	1	0.98	0.98
1189	94.2	93.2	92.5	83.0	90.9	0.89	0.91	0.89	0.82
25PDB	94.8	92.3	87.0	86.4	90.3	0.88	0.89	0.87	0.84

### 2.2. Performance Comparison with Existing Methods

In order to evaluate the effectiveness of the proposed method, we first compare it with the other existing methods based on the Z277 and Z498 datasets. The results from the jackknife tests are summarized in Table 2 and Table 3.

**Table 2:** Comparison of different methods by the jackknife test for the Z277 dataset.

Method	Prediction Accuracy (%)				
	All-α	All-β	α/β	α + β	Overall
Neural network [7]	68.6	85.2	86.4	56.9	74.7
Component coupled [23]	84.3	82.0	81.5	67.7	79.1
LogitBoost [19]	81.4	88.5	92.6	72.3	84.1
IGA-SVM [10]	84.3	88.5	92.6	70.7	84.5
CWT-PCA-SVM [27]	85.7	90.2	87.7	80.1	85.9
Markov-SVM [42]	90.0	85.2	86.4	81.5	85.9
SVM fusion [21]	85.7	90.2	93.8	80.0	87.7
AAC-PSSM-AC [30]	88.6	95.1	97.5	81.5	91.0
Our method	97.1	98.4	97.5	96.9	97.5

**Table 3:** Comparison of different methods by the jackknife test for the Z498 dataset.

Method	Prediction Accuracy (%)				
	All-α	All-β	α/β	α + β	Overall
Neural network [7]	86.0	96.0	88.2	86.0	89.2
Component-coupled [23]	93.5	88.9	90.4	84.5	89.2
SVM fusion [21]	99.1	96.0	80.9	91.5	91.4
Markov-SVM [42]	91.6	94.4	96.3	91.5	93.6
IGA-SVM [10]	96.3	93.6	97.8	89.2	94.2
LogitBoost [19]	92.6	96.0	97.1	93.0	94.8
CWT-PCA-SVM [27]	94.4	96.8	97.0	92.3	95.2
AAC-PSSM-AC [30]	94.4	96.8	97.8	93.8	95.8
Our method	98.1	100	98.5	97.7	98.6

As is shown, our method obtains the overall accuracies of 97.5% and 98.6% on these two datasets, which are better than the other classifiers including neural network [7], component-coupled [23], LogitBoost [19], AAC-PSSM-AC [30] and SVM-based methods [10,21,27,42]. It is worth noting that the AAC-PSSM-AC algorithm, which extracts AAC and AC features solely from the PSSM profile to represent a protein, also attains the second best prediction performance. This illustrates that the PSSM profile indeed provides important and useful discriminatory information for predicting protein structural class. In addition, we notice that the total accuracies of our method are higher than those of the LogitBoost and SVM fusion classifiers, which incorporate many weak classifiers to construct a strong classifier. This suggests that designing better sequence representations is more important than exploring more complex classifiers.

To explore the impact of sequence similarity on the performance of our method, we make comparisons with other competing prediction methods against two low-similarity datasets (*i.e.*, 1189 and 25PDB). The high prediction accuracies of these methods are mainly due to extracting features from the PSSM profile as well as the predicted secondary structure information. The approaches based on PSSM include AADP-PSSM [31], AAC-PSSM-AC [30], Comb_11,10,6 [22], LCC-PSSM [29] and PSSM-SPINE-S [34]. The approaches based on the predicted secondary structure include SCPRED [9], RKS-PPSC [43], MODAS [33], and PSSM-SPINE-S [34]. The results by jackknife tests are listed in Table 4 and Table 5.

**Table 4:** Performance comparison of different methods on the 1189 dataset.

Method	Prediction Accuracy (%)				
	All-α	All-β	α/β	α + β	Overall
AADP-PSSM [31]	69.1	83.7	85.6	35.7	70.7
AAC-PSSM-AC [30]	80.7	86.4	81.4	45.2	74.6
Comb_11,10,6 ^1^ [22]	80.2	83.6	85.4	44.6	74.8
SCPRED [9]	89.1	86.7	89.6	53.8	80.6
LCC-PSSM [29]	89.2	88.8	85.6	58.5	81.2
RKS-PPSC [43]	89.2	86.7	82.6	65.6	81.3
MODAS [33]	92.3	87.1	87.9	65.4	83.5
PSSM-SPINE-S [34]	98.2	91.5	83.8	72.2	86.3
Our method	94.2	93.2	92.5	83.0	90.9

^1^ The result is evaluated using 10-fold cross-validation test.

**Table 5:** Performance comparison of different methods on the 25PDB dataset.

Method	Prediction Accuracy (%)				
	All-α	All-β	α/β	α + β	Overall
AADP-PSSM [31]	83.3	78.1	76.3	54.4	72.9
AAC-PSSM-AC [30]	85.3	81.7	73.7	55.3	74.1
Comb_11,10,6 ^1^ [22]	86.1	80.8	80.6	60.1	76.7
LCC-PSSM [29]	91.7	80.8	79.8	64.0	79.0
SCPRED [9]	92.6	80.1	74.0	71.0	79.7
MODAS [33]	92.3	83.7	81.2	68.3	81.4
RKS-PPSC [43]	92.8	83.3	85.8	70.1	82.9
PSSM-SPINE-S [34]	96.8	93.7	90.1	87.0	92.2
Our method	94.8	92.3	87.0	86.4	90.3

^1^ The result is evaluated using 10-fold cross-validation test.

For the 1189 dataset, the proposed method outperforms all other methods listed in Table 4, with an accuracy of 90.9%. It is also shown that studies which relied on predicted secondary structure to enhance the accuracy could not reach a result too much better than 80%. This may be due to the limited accuracy (about 80%) of the predicted secondary structure by PSIPRED. Referring to Table 5, the overall accuracy of our method achieves 90.3% for the 25PDB dataset, which is higher than those of other methods except for PSSM-SPINE-S. It should be pointed out that PSSM-SPINE-S combines PSSM features with secondary structure features extracted from the SPINE-X [44] to improve the performance. This indicates that predicted secondary structure information plays an important complementary role for predicting protein structural class. However, the proposed representation also attains satisfactory performance when only the PSSM profile is employed. 

From the above comparisons, our method shows substantial improvements for the prediction of protein structural class. This could be attributed to the informative feature extraction technique based on GapDPC computed directly from PSSM and selected optimal features by SVM-RFE.

## 3. Materials and Methods

### 3.1. Datasets

Two datasets (*i.e.*, Z277 and Z498) constructed by Zhou [23] are first used to evaluate the proposed method, and they contain 277 and 498 protein domains, respectively. Despite the relatively small size of these two datasets, they were widely used in many studies. To explore the impact of the proposed method on the low-similarity datasets, another two datasets, 1189 [14] and 25PDB [15], are also studied separately. The first one consists of 1092 protein domains with sequence similarity less than 40% and the second one includes 1673 protein domains with sequence similarity lower than 25%. The detailed compositions of four datasets are listed in Table 6.

**Table 6:** The compositions of four datasets adopted in this study.

Dataset	All-α	All-β	α/β	α + β	Total
Z277	70	61	81	65	277
Z498	107	126	136	129	498
1189	223	294	334	241	1092
25PDB	443	443	346	441	1673

### 3.2. Protein Sequence Representation

Previous successful applications of PSSM profile illustrate that evolutionary information is more informative than sequence itself [28,30]. In this section, a simple sequence representation which combines PSSM profile and the concept of GapDPC is developed for the proposed prediction method.

The profile of each sequence is generated by running PSI-BLAST program [45] against the NCBI’s non-redundant (NR) database with three iterations and a cutoff *E*-value of 0.001. The (*i*, *j*)th entry of the resulting matrix represents the probability of amino acid type *j* occurring at the *i*th position of the query sequence. The PSSM elements are mapped to the range of (0, 1) by the following sigmoid function:

(1) $$f\left(x\right)=\frac{1}{1+e^{-x}}$$

where *x* is the original PSSM value.

For convenience, let us denote

(2) $$P=\left(P_{1},\;P_{2},\ldots ,P_{20}\right)$$

as the PSSM of the query sequence *S*, where

(3) $$P_{j}={\left(p_{1,j},\;p_{2,j},\ldots ,p_{L,j}\right)}^{T}\;\left(j=1,2,\ldots ,20\right)$$

*L* is the length of the query sequence *S*, and *T* is the transpose operator.

Since the structural class of a protein is closely related to its dipeptide composition (DPC) [31], we first extend the concept of traditional DPC from the primary sequence to the PSSM. DPC is defined as a 400-dimentional vector:

(4) $$X=\left(x_{1,1},\;\ldots ,x_{1,20},x_{2,1},\;\ldots ,x_{2,20},\ldots ,x_{20,1},\;\ldots ,x_{20,20}\right)$$

where

(5) $$x_{i,j}=\overset{L-1}{\underset{k=1}{\sum }}p_{k,i}\times p_{k+1,j}\;\left(1\leq i,j\leq 20\right)$$

As we all know, sequence-order information is as important as its residue composition in a protein sequence. To partially reflect the local sequence-order effect, GapDPC is introduced to explore the long-range correlation between two residues separated by one or more positions, which can be calculated by

(6) $$y_{i,j,g}=\overset{L-g-1}{\underset{k=1}{\sum }}p_{k,i}\times p_{k+g+1,j}\;\left(1\leq i,j\leq 20\right)$$

where *g* is the distance between amino acid *i* and amino acid *j*. Note that GapDPC is reduced to DPC when *g* is equal to 0.

These elements of the three-dimensional matrix *y_i,j,g_*, which correspond to the frequencies of PSSM-based gapped-dipeptides, are used to represent the given query sequence. We generate PSSM-based GapDPC for *g* = 0, 1, 2, … , *G*, which results in 400*(*G* + 1) features for each sequence.

### 3.3. Recursive Feature Selection

After running the proposed feature extraction technique, all protein sequences with different length are converted into numerical feature vectors with the same dimension. In order to decrease feature redundancy and reduce computation cost, we introduce a recursive feature selection approach to rank the features according to their importance. Support vector machine-recursive feature elimination (SVM-RFE), which was originally carried out on gene selection for cancer classification by Guyon and his co-workers [46], has been proven to be an effective tool for dimensionality reduction in the field of pattern recognition. The process is conducted as follows. First, all the feature vectors of proteins for each dataset are trained using SVM with a linear kernel. Then, the features are ranked with decreasing order according to their weights which reflect the relevance to prediction of protein structural class. Finally, top *K* features with the most relevant ranks are selected to represent each protein sequence.

### 3.4. Support Vector Machine

SVM, which is first introduced by Vapnik [47], is considered as the state-of-the-art machine learning algorithm for classification. It maps the input data into higher dimensional feature space using the kernel function and then finds an optimal hyper-plane to separate a given set of labeled data. Among a lot of classification algorithms used for prediction of protein structural class, SVM has shown the best prediction accuracies [9]. In this work, the SVM classifier implemented by the LIBSVM software (Chang and Lin, Taipei, Taiwan) [48] is employed to perform the prediction. Though LIBSVM provides four basic kernel functions, *i.e.*, linear, polynomial, radial basis function (RBF) and Gaussian, RBF kernel is adopted here due to its better performance than other kernel functions. The cost parameter *C* and the width parameter γ are optimized based on the grid search algorithm implemented in the LIBSVM software.

### 3.5. Cross-Validation and Performance Evaluation

In this study, the jackknife test is adopted to evaluate the prediction performance of our method. Although the jackknife test is time-consuming, it is considered more objective than other cross-validation methods (e.g., independent dataset test and sub-sampling test) [49]. The basic idea behind the jackknife test lies in systematically calculating the statistic estimate, leaving out each sample from a dataset and then finding the average of these calculations. To evaluate the performance of our predictor, the accuracy, overall accuracy and Matthews correlation coefficient (MCC) are adopted as the comparative measures. They are defined by the following formulas:

(7) $$Accuracy_{j}=\frac{TP_{j}}{TP_{j}+FN_{j}}=\frac{TP_{j}}{\left|C_{j}\right|}$$

(8) $$MCC_{j}=\frac{TP_{j}\times TN_{j}-FP_{j}\times FN_{j}}{\sqrt{\left(TP_{j}+FP_{j}\right)\left(TP_{j}+FN_{j}\right)\left(TN_{j}+FP_{j}\right)\left(TN_{j}+FN_{j}\right)}}$$

(9) $$Overall\;accuracy=\frac{\sum_{j}TP_{j}}{\sum_{j}\left|C_{j}\right|}$$

where *TP_j_*, *TN_j_*, *FP_j_*, *FN_j_*, and |*C_j_*| are the number of true positives, true negatives, false positives, false negatives, and proteins in the structural class *C_j_*, respectively.

## 4. Conclusions

In this study, we combine gapped-dipeptides with SVM-RFE to predict protein structural class. In order to partly reflect the local sequence-order effect, the proposed method extracts features from gapped-dipeptides of various distances based on PSSM. These features are further ranked by SVM-RFE according to their importance and the optimal features are input to SVM classifiers to perform the prediction. Comparison with other existing techniques on four benchmark datasets indicates that our predictor is a useful tool to predict protein structural class and also shows the generality of the proposed method.
